# New Appliances and Things Medical

**Published:** 1900-11-10

**Authors:** 


### NEW APPLIANCES AND THINGS MEDICAL.

[We shall be glad to receive, at our Office, 23 & 29 Southampton Street, Strand, London, W.C., from the manufacturers, specimens of all new preparations and appliances which may be brought out from time to time.]

### PROTECTOR CARBOLIC SOAP.

(United Alkali Company Limited, Hazlehurst's Camden Soap Works, Runcorn.)

We have examined a sample of the above carbolic soap, and regard it as eminently suitable for household purposes, such as the scrubbing of floors, stairs, walls, &c. Antiseptics in themselves are doubtless of value in destroying the living germs of disease. But cleanliness is far more destructive and inimical to bacterial life. The full use of a soap with such properties as those possessed by "Protector Carbolic Soap" will serve as a stronger defence against the spread of infectious disease than far severer measures, which only comprise cleanliness or disinfection separably and not combined. The soap basis of this preparation appears of sound manufacture: it lathers freely, is economical in price and in use, and being combined with a liberal percentage of carbolic acid it must be regarded as a powerful antiseptic.

### TABLOIDS OF FERRICHTHOL AND ICHTHYOL CALCII.

(Gustav Hermanni, 20 High Holborn, London, W.C.)

We have had occasion to mention various ichthyol preparation in several issues of this journal: the two preparations under review at the moment are comparatively new combinations of this valuable drug. The iron preparation, or Ferrichthol, has now had a sufficiently extended trial in cases of anæmia to warrant its acceptance by medical men as an assimilable form of iron with no irritating action on the mucous lining of the alimentary tract. The exact action of Ichthyol is apparently unknown: its results are, however, definite; it has a very marked action on tissue metabolism, and especially on the healthy action of the skin, whether locally applied or taken by the mouth. Ferrichthol is especially indicated, therefore, in cases of anæmia combined with disorders of the skin, such as eczema and dermatitis. Ichthyol appears to be a slight antiseptic agent, but its efficiency is probably rather due to its stimulating properties, whereby the tissues are put in a condition of effectual resistance to microbic invasion. The Ichthyol Calcii may be used under similar conditions to those in which the iron preparation are indicated, but its chief application appears to be the treatment of bone diseases due to constitutional dyscrasies such as rachitis, scrofula, rheumatic conditions, &c. It should be given in tabloid form (1½ grain) to the extent of about 10 grains in the day.

### PROTENE PREPARATIONS.

(Protene Company, 36 Welbeck Street, London, W.)

We have before spoken highly of the preparations made by this firm. The flour which forms the basis of all their specialities, whether bread, biscuits, or cakes, consists chiefly of a proteid substance prepared from milk. For those persons who are forbidden to indulge in farinaceous or carbohydrate foods these protene preparations are a real boon. They are exceedingly nice, and although they cannot be said to be exactly like their farinaceous prototypes in taste and flavour, they are a very good imitation. Diabetics and others thoroughly appreciate the great variety which is afforded by these starch-free foods.

